# Facile fabrication of super-hydrophobic nano-needle arrays via breath figures method

**DOI:** 10.1186/1556-276X-6-616

**Published:** 2011-12-06

**Authors:** Jiseok Kim, Brian Lew, Woo Soo Kim

**Affiliations:** 1 Mechatronic Systems Engineering, School of Engineering Science, Simon Fraser University, 250-13450 102nd Avenue, Surrey, BC V3T 0A3, Canada

**Keywords:** super-hydrophobic, nano-needle, honeycomb, anisotropic pattern

## Abstract

Super-hydrophobic surfaces which have been fabricated by various methods such as photolithography, chemical treatment, self-assembly, and imprinting have gained enormous attention in recent years. Especially 2D arrays of nano-needles have been shown to have super-hydrophobicity due to their sharp surface roughness. These arrays can be easily generated by removing the top portion of the honeycomb films prepared by the breath figures method. The hydrophilic block of an amphiphilic polymer helps in the fabrication of the nano-needle arrays through the production of well-ordered honeycomb films and good adhesion of the film to a substrate. Anisotropic patterns with water wettability difference can be useful for patterning cells and other materials using their selective growth on the hydrophilic part of the pattern. However, there has not been a simple way to generate patterns with highly different wettability. Mechanical stamping of the nano-needle array with a polyurethane stamp might be the simplest way to fabricate patterns with wettability difference. In this study, super-hydrophobic nano-needle arrays were simply fabricated by removing the top portion of the honeycomb films. The maximum water contact angle obtained with the nano-needle array was 150°. By controlling the pore size and the density of the honeycomb films, the height, width, and density of nano-needle arrays were determined. Anisotropic patterns with different wettability were fabricated by simply pressing the nano-needle array at ambient temperature with polyurethane stamps which were flexible but tough. Mechanical stamping of nano-needle arrays with micron patterns produced hierarchical super-hydrophobic structures.

**PACS**: 05.70.Np, *68.55.am*, *68.55.jm*

## Background

Super-hydrophobic surfaces have been designed to study scientific fundamentals of water repellency and to use them for practical applications such as self-cleaning materials [1,2], micro-fluidics [3], nano-imprinting stamps [4], and biotechnology [5]. It has been well known that super-hydrophobic surfaces can be fabricated by controlling roughness on hydrophobic materials [6,7]; thus, both top-down [8-12] and bottom-up [13-16] methods have been applied to make the surfaces of hydrophobic materials rough in micro- and nanoscales to enhance their hydrophobicity.

A 2D array of hexagonally packed nano-needles has been introduced to present super-hydrophobicity. Chen et al. have fabricated a 2D array of ZnO needles using polystyrene [PS] microspheres as a template and electroplating ZnO on the PS template [17]. The fabricated array showed super-hydrophobicity. Yabu et al. [14] also reported a nano-needle array fabricated by peeling off the top portion of the honeycomb films which were prepared by a designed fluorinated polymer using the breath figures method. For the breath figures method, a highly organized, hexagonal thin film, called a honeycomb film, is induced by the evaporation of organic solvent after water droplets sink into the polymer solution. Then, the top surface of the prepared honeycomb film can be simply taped off and the nano-needle array is formed on the film.

Honeycomb-structured thin films have been usually fabricated by the breath figures method with a polymer like PS [18]. Surfactants or terminal-modified PS have been used to obtain more regular honeycomb structures because they can act to stabilize water droplets condensed in the polymer solution in which the polymer is dissolved in an organic solvent during the breath figures method [18-20]. On the other hand, one problem to consider when generating the nano-needle array is that PS is not adhesive to conventional substrates such as glass and Si. Good substrate adhesion is important when the top portion of the honeycomb film is removed because good adhesion can ensure that the entire honeycomb film is not detached from the substrate. Thus, amphiphilic block copolymers which have hydrophobic and hydrophilic polymers together are advantageous for making well-ordered honeycomb films and also the nano-needle array because there is no need to add another surfactant for stabilizing the water droplets in the polymer solution; in addition, the substrate adhesion problem can be solved with the hydrophilic block. The hydrophilic block can stabilize water droplets in the polymer solution and also attach firmly to inorganic substrates such as glass or Si as well as flexible substrates such as poly(ethylene terephthalate) [PET] or poly(ethylene naphthalate). In this study, polystyrene-*block*-poly (2-vinyl pyridine) [PS-*b*-P2VP] has been used to fabricate honeycomb film and nano-needle arrays. PS (surface tension, ~33 mN/m) consists of a hydrophobic block, while P2VP (surface tension, ~60 mN/m) plays a role as a hydrophilic block.

Anisotropic patterns which have wettability difference with hydrophobicity and hydrophilicity together have been widely applied to selective cell growth [5], water collection [21], micro-fluidic channels [3], and templates for patterning [22,23]. To fabricate patterns with wettability difference, several methods have been used, such as photolithography [5,22,23] and chemical treatment [3,21]. Hot embossing has also been used to make a hierarchical pattern with a honeycomb structure [24]. However, these are not simple and require fancy instruments or harsh chemicals with a high temperature. In this study, we made the anisotropic pattern with wettability difference formed by simply pressing the nano-needle array with flexible polyurethane [PU] stamps at ambient temperature.

## Methods

### Materials

The diblock copolymer PS-*b*-P2VP (27,000-*b*-4,000 g/mol, *φ*_P2VP _= 13.4%) was purchased from Polymer Source Inc. (Dorval, QC, Canada). Carbon disulfide (CS_2_) was obtained from Sigma-Aldrich Inc. (St. Louis, MO, USA). PS-*b*-P2VP was dissolved at 0.25, 0.5, 1, and 2 wt.% in the PS-selective solvent (CS_2_).

### Preparation of honeycomb films using the breath figures method

The block copolymer solutions were drop-cast onto several substrates such as PET, glass, or Si inside an acrylic glass chamber at room temperature. Construction of the chamber provided a relatively closed system through which humidity could be kept constant during the course of the experiment. Humidity fluctuations were kept within 90-95%. Humid air was pumped into the chamber until an appropriate relative humidity was reached. Airflow was reduced to eliminate macroscopic convection currents and other unpredictable thermodynamic consequences, but kept high enough to maintain the desired humidity. This allowed homogeneous honeycomb patterning with a relatively large coverage. After 5 min, the solvent evaporated and the slides were removed from the chamber to allow water evaporation under ambient conditions. The films obtained were circular, with diameters of around 3 cm.

### Preparation of nano-needle array by a simple taping-off method

Adhesive Scotch tape was placed on the surface of the honeycomb films in order to remove the top portion of the polymer thin film. The surface of the tape was rubbed smoothly with the thumb to ensure full contact with the top layer of the honeycomb film without trapped air. Then, the tape was peeled off slowly. The prepared nano-needle arrays were brought into analysis and stamping.

### Fabrication of patterns with different wettability by PU stamps

First, a patterned Si master on which PU stamps would be replicated was fabricated by conventional photolithography with a photo-mask. PU stamps were fabricated by replication of the pre-patterned master with a UV-curable urethane acrylate prepolymer. Urethane acrylate (EBECRYL resin, Cytec Industries, Woodland Park, NJ, USA) dissolved into propylene glycol methyl ether acetate (Sigma-Aldrich Inc., St. Louis, MO, USA) and 5wt.% photo-initiator (Irgacure 184, CIBA, Tarrytown, NY, USA) was added to the solution. The solution was then dropped on a substrate, which afterwards was covered with the patterned Si master. The sample was then UV-cured at 1 J/cm^2 ^with 350-nm wavelength UV lamp. Then, PU stamps with pre-designed negative patterns were placed on the prepared nano-needle array and were pressed with slight pressure < 1 N/m^2 ^by hand for 5 min at ambient temperature.

### Characterization

Atomic force microscopic images and the height profiles of the honeycomb films and the corresponding nano-needle array were obtained by PARK systems XE-100 AFM. Optical microscopy was performed using a Nikon Eclipse LV100 with a 50-W halogen light source, and images were obtained via Nikon's digital sight camera system. Scanning electron microscope [SEM] images were obtained via an FEI DualBeam Strata 235 in 5.00 kV. Samples were coated with gold prior to SEM imaging.

Contact angles of water (surface energy, 72.75 mN/m at 20°C) on the surfaces were measured by the sessile drop technique. Of the water, 50 μl was dropped on each surface and each contact angle measured by a contact angle goniometer.

## Results and discussion

### Fabrication of honeycomb films and nano-needle arrays

**Figure 1:**
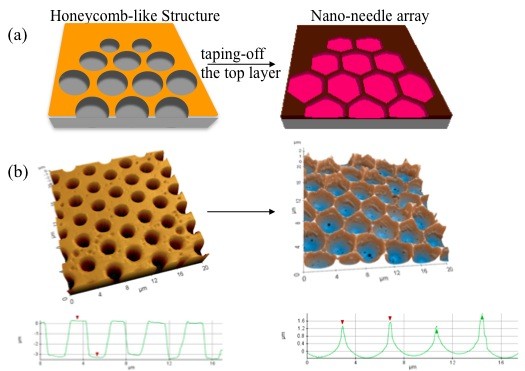
**Nano-needle formation from honeycomb film**. (**a**) Schematic diagram of process of fabricating a nano-needle array. (**b**) AFM topographical images of a honeycomb film and a nano-needle array.

During the breath figures method, due to the high humidity within the closed chamber, water droplets were condensed on the surface of the polymer solution. To maintain the spherical orientation of the droplets over the solution, the breath figures method requires immiscibility between the solvent and the water [25]. Subsequent evaporation of the solvent and then the water leaves behind an imprint of the water droplet on the block copolymer film. This results in an ordered honeycomb film, as shown on the left of Figure 1a. After removing the top portion of the honeycomb film by simply putting an adhesive tape on the film and peeling it off, a 2D array of nano-needles was revealed as sharp tips with about 10-nm radius of curvature were formed at the vertices of hexagons, as shown schematically on the right of Figure 1a. The atomic force microscopic [AFM] images corresponding to Figure 1a are shown in Figure 1b. Circular shapes on the honeycomb film became hexagonal because the tension from the top layer had been removed. After taping off the top layer, the pore depth was reduced from 3.5 to 2.0 μm, according to the profile graphs generated from the AFM images in Figure 1b. The formation of the nano-needles with sharp tips could be confirmed by the brighter spots at the vertices of the hexagon on the right AFM image in Figure 1b.

As mentioned in the "Background," PS has been used to fabricate honeycomb films with the use of surfactants which have been added to provide water droplets with stability during the breath figures method. PS was not firmly attached to conventional substrates such as glass and Si because it is hydrophobic. Thus, nano-needles were hardly formed without all the film detached from the substrate when PS honeycomb films prepared on glass substrates were peeled off by a Scotch tape in our trials. In this respect, the use of an amphiphilic block copolymer was very helpful in fabricating honeycomb films and nano-needle arrays using the simple taping-off method. A hydrophilic block, P2VP, played both roles as a surfactant for the stability of water droplets and a substance for firm attachment to the substrate.

### Characteristics of nano-needle arrays according to honeycomb pore density

**Figure 2:**
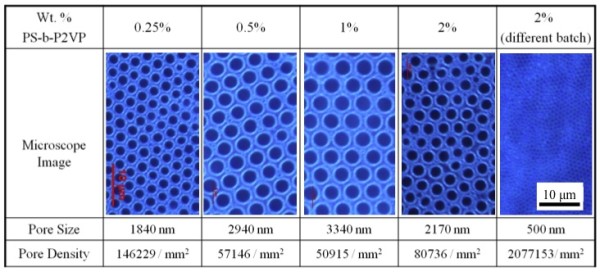
**Honeycomb structure**. Controlled honeycomb structure with respect to pore size and pore density depending on polymer concentration.

**Figure 3:**
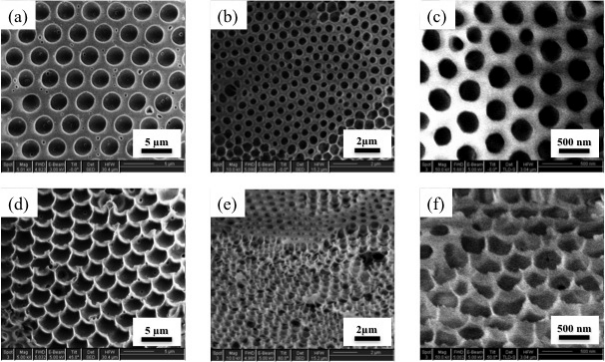
**Scanning electron microscopy**. SEM images with controllable sizes of honeycomb structures (**a**, **b**, **c**) and nano-needles (**d**, **e**, **f**).

As shown in Figure 2, we have obtained honeycomb-structured films with pores which show a uniform distribution of size over about 100 cm^2^. The size of the pores on the honeycomb films appears to increase up to a certain concentration before decreasing, as shown in Figure 2. Meanwhile, the pore density decreases up to a certain concentration before increasing. This indicates that the size of the water droplets during the fabrication of the honeycomb films by the breath figures method is not equal in each case; it increases with concentration and then decreases, following the pore density trend. This might result from the restriction on the growth of the water droplets and the degree of water droplet sinking into the polymer solution as the polymer concentration increases. In addition, polymer solution from different polymer batches with the same composition showed the reduced pore size at the same polymer concentration (2 wt.%), as shown in Figure 2. This might be caused by a slight difference in composition between polymer batches. Although the exact mechanism of pore size variation is yet to be determined, honeycomb films with various pore sizes and densities have been fabricated. As a result, the density of nano-needles formed by peeling off the top layer of the honeycomb films might be controlled. Figure 3 shows representative SEM images of nano-needles generated from the honeycomb films with different pore densities. Figure 3a, d shows the honeycomb and the corresponding nano-needle array with 3.5-μm pore diameter. Similarly, pore sizes of 1 μm and 500 nm are shown in Figure 3b, e and Figure 3c, f, respectively.

### Super-hydrophobicity presented on nano-needle arrays

**Figure 4:**
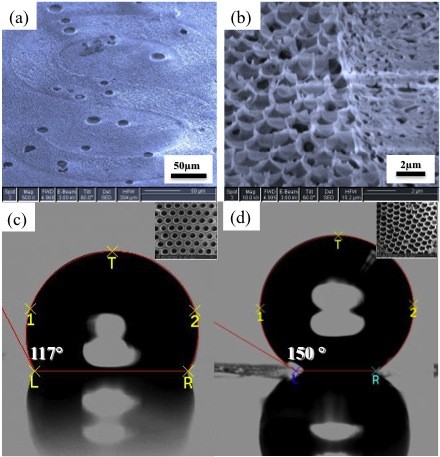
**Hierarchical super-hydrophobic pattern**. (**a**) Stamped letter "S". (**b**) Interface of the stamped and unstamped area of the nano-needle array. (**c**) Contact angle of the stamped area. (**d**) Contact angle of the unstamped area with nano-needles.

Hydrophobicity is related to the water contact angle; for pure PS, the angle is around 90° [26]; the contact angle for P2VP is 55° [27]. The PS-*b*-P2VP block copolymer has blocks of unequal hydrophobicity, with P2VP being relatively more hydrophilic than PS. Therefore, it may be energetically favorable for a P2VP layer to associate with the water droplet while PS associates with the CS_2 _solvent and air. Therefore, the surface of the honeycomb film should be hydrophobic in nature because the PS layer forms on the surface of the honeycomb film where the film contacts air. It could be confirmed by a contact angle which is measured to be 117° for the honeycomb film before the taping-off procedure, as shown in Figure 4b. The reason for the contact angle being more than 90° is that the honeycomb film has some roughness compared with the flat surface of PS. Other block copolymers have also shown the same result as a honeycomb film has a larger contact angle than a flat film does [14].

Nano-needle arrays prepared from the honeycomb film with a pore size of 3.5 μm showed an average contact angle of 150°, which means super-hydrophobicity (Figure 4b). When prepared with a smaller pore size (500 nm), the array of nano-needles showed a lower average contact angle of about 145°. Topographical characteristics such as width and radius of curvature of nano-needles appear to be properties which determine the hydrophobicity of rough surfaces [28], although these should be further analyzed. According to Draper et al. [27], the parameters related to the hydrophobicity of rough surfaces are deduced from the following equations:

$D*=1+\frac{D}{R}$, $H*=\frac{2\left(1-\cos\theta \right)\times R\times l_{\text{cap}}}{D^{2}}$, where $l_{\text{cap}}={\left(\frac{\gamma_{\text{lv}}}{\rho g}\right)}^{0.5}$ is the capillary length (*D *being half of the peak-to-peak width of the re-entrant pattern, *R *the curvature at the peak of the pattern, *θ *the contact angle on a flat surface, *γ*_lv _the surface energy of the liquid, *ρ *the density of the liquid, and *g *the gravitational acceleration).

The above equations give us the parameters for a contact angle (D*) and the robustness of a meta-stable Cassie state (H*). As parameter D* increases, a fraction of solid/liquid, that is, the surface/water contact, decreases and thus the contact angle increases. As parameter H* increases, the robustness of the state increases and the contact angle remains high. For the nano-needle array with 3.5-μm diameter, *R *is 10 nm, *D *is 3.5 μm, *θ *is 90° for flat PS film, and *l*_cap _is 2.72 mm. D* and H* were calculated to be 351 and 2.11, respectively.

For the nano-needle array with 500-nm diameter, D* and H* were 51 and 14.8, respectively. This might explain why the nano-needle array with a smaller pore diameter has a smaller contact angle (145° for 500 nm compared with 150° for 3.5 μm) - the difference in D* is large but that in the contact angle is not because D* does not contain anything related to material property but to structural property, and thus, it can only provide a relative measure of the contact angles - from the fact that the nano-needle array with 1-μm pore diameter has D* smaller than that with 3.5-μm pore diameter. Therefore, the width and radius of curvature of nano-needles might be customized to obtain better super-hydrophobicity. Therefore, this simple method, which includes drop casting and the breath figures method of the amphiphilic block copolymer solution and taping-off of the resulting honeycomb film, would be advantageous for the fabrication of super-hydrophobic surfaces with respect to cost and ease of fabrication.

### Patterns with different wettability fabricated by simple pressing with PU stamps

Anisotropic patterns with water wettability difference were formed simply by pressing nano-needle array with a PU stamp. It has been revealed by flattening the nano-needle array that the flattened array showed a small value of contact angle compared with the nano-needle array. The pattern of the PU stamp which had lines or alphabet letters has been successfully stamped onto the nano-needle array. One of the stamped patterns is shown in Figure 4b. As shown on the right of Figure 4a, the pressed area (the right side) is smooth and flat, in contrast to the unpressed area (the left side). Thus, the unpressed area in which nano-needles remain unaffected would be super-hydrophobic, while the pressed area in which nano-needles are made flat would show a much decreased hydrophobicity or a little of hydrophilicity due to the hydrophilic P2VP block. This anisotropic pattern with different wettability will be further applied as a template for electrical materials such as metals and semiconductors which are selectively deposited on the hydrophilic area.

## Conclusions

A 2D array of nano-needles has been fabricated by simply taping off the top portion of honeycomb films prepared by drop casting on various substrates like glass or PET and subsequently by applying the breath figures method. The use of an amphiphilic block copolymer, PS-*b*-P2VP, enabled fabricating well-ordered honeycomb structures stabilizing water droplets formed in the polymer solution during the breath figures method and easy peeling off of the top portion of the honeycomb film resulting from a strong adhesion of the polymer thin film to the substrates.

The 2D array of nano-needles had about 10-nm radius of curvature and showed super-hydrophobicity as the average water contact angle on the nano-needle array was measured to be 150°. The pore size and the density of the honeycomb film could be controlled by polymer concentration and polymer micelle formation. In this respect, the height, width, and density of nano-needles on the nano-needle array would be controlled as well. The characteristics of nano-needles were expected to affect hydrophobicity due to the fact that nano-needle arrays prepared from honeycomb films with different pore sizes and densities showed different water contact angles.

Anisotropic patterns with different water wettability were fabricated by simply pressing the nano-needle array with flexible PU stamps by hand at ambient temperature. The anisotropic pattern will be further used as a template to pattern electrical materials such as metals or semiconductors.

## Competing interests

The authors declare that they have no competing interests.

## Authors' contributions

WSK designed and directed this study. JK and BL together carried out the fabrication and characterization of the honeycomb films and nano-needle arrays, and wrote the paper. JK also fabricated PU stamps and carried out stamping of the nano-needle arrays. All authors read and approved the final manuscript.

## References

[B1] Blossey R Self-cleaning surfaces - virtual realities Nat Mater 2003 2 301 306 10.1038/nmat856 12728235

[B2] Cheng YT Rodak DE Wong CA Hayden CA Effects of micro- and nano-structures on the self-cleaning behaviour of lotus leaves Nanotechnology 2006 17 1359 1362 10.1088/0957-4484/17/5/032

[B3] Mumm F van Helvoort ATJ Sikorski P Easy route to superhydrophobic copper-based wire-guided droplet microfluidic systems ACS Nano 2009 3 2647 2652 10.1021/nn900607p 19681579

[B4] Kim WS Jin JH Bae BS Low adhesive force of fluorinated sol-gel hybrid materials for easy de-moulding in a UV-based nano-imprint process Nanotechnology 2006 17 1212 1216 10.1088/0957-4484/17/5/008

[B5] Ishizaki T Saito N Takai O Correlation of cell adhesive behaviors on superhydrophobic, superhydrophilic, and micropatterned superhydrophobic/superhydrophilic surfaces to their surface chemistry Langmuir 2010 26 8147 8154 10.1021/la904447c 20131757

[B6] Reinhoudt D Li XM Crego-Calama M What do we need for a superhydrophobic surface? A review on the recent progress in the preparation of superhydrophobic surfaces Chem Soc Rev 2007 36 1350 1368 10.1039/b602486f 17619692

[B7] Yang C Tartaglino U Persson BNJ Influence of surface roughness on superhydrophobicity Phys Rev Lett 2006 97 116103 17025908 10.1103/PhysRevLett.97.116103

[B8] Shiu JY Kuo CW Chen PL Mou CY Fabrication of tunable superhydrophobic surfaces by nanosphere lithography Chem Mater 2004 16 561 564 10.1021/cm034696h

[B9] Gao D Cao LL Hu HH Design and fabrication of micro-textures for inducing a superhydrophobic behavior on hydrophilic materials Langmuir 2007 23 4310 4314 10.1021/la063572r 17371061

[B10] Yang SM Park SG Lee SY Jang SG Perfectly hydrophobic surfaces with patterned nanoneedles of controllable features Langmuir 2010 26 5295 5299 10.1021/la100409c 20297829

[B11] Yang SM Park SG Moon HH Lee SK Shim J Bioinspired holographically featured superhydrophobic and supersticky nanostructured materials Langmuir 2010 26 1468 1472 10.1021/la9035826 19928976

[B12] Zhao H Law KY Sambhy V Fabrication, surface properties, and origin of superoleophobicity for a model textured surface Langmuir 2011 27 5927 5935 10.1021/la104872q 21486088

[B13] Ajayaghosh A Srinivasan S Praveen VK Philip R Bioinspired superhydrophobic coatings of carbon nanotubes and linear pi systems based on the "bottom-up" self-assembly approach Angew Chem Int Edit 2008 47 5750 5754 10.1002/anie.200802097 18604864

[B14] Yabu H Takebayashi M Tanaka M Shimomura M Superhydrophobic and lipophobic properties of self-organized honeycomb and pincushion structures Langmuir 2005 21 3235 3237 10.1021/la050013w 15807559

[B15] Feng L Song YL Zhai J Liu BQ Xu J Jiang L Zhu DB Creation of a superhydrophobic surface from an amphiphilic polymer Angew Chem Int Edit 2003 42 800 802 10.1002/anie.200390212 12596204

[B16] Genzer J Efimenko K Creating long-lived superhydrophobic polymer surfaces through mechanically assembled monolayers Science 2000 290 2130 2133 10.1126/science.290.5499.2130 11118144

[B17] Chen L-Y Lai C-H Wu P-W Fan S-K Electrowetting of superhydrophobic ZnO inverse opals J Electrochem Soc 2011 158 P93 P99 10.1149/1.3594723

[B18] Bunz UHF Breath figures as a dynamic templating method for polymers and nanomaterials Adv Mater 2006 18 973 989 10.1002/adma.200501131

[B19] Xu ZK Wan LS Ke BB Li XK Meng XL Zhang LY Honeycomb-patterned films of polystyrene/poly(ethylene glycol): preparation, surface aggregation and protein adsorption Sci China Ser B 2009 52 969 974 10.1007/s11426-009-0007-1

[B20] Yunus S Delcorte A Poleunis C Bertrand P Bolognesi A Botta C A route to self-organized honeycomb microstructured polystyrene films and their chemical characterization by ToF-SIMS imaging Adv Funct Mater 2007 17 1079 1084 10.1002/adfm.200600470

[B21] Zhai L Berg MC Cebeci FC Kim Y Milwid JM Rubner MF Cohen RE Patterned superhydrophobic surfaces: toward a synthetic mimic of the Namib Desert beetle Nano Lett 2006 6 1213 1217 10.1021/nl060644q 16771582

[B22] Lai YK Huang JY Gong JJ Huang YX Wang CL Chen Z Lin CJ Superhydrophilic-superhydrophobic template: a simple approach to micro- and nanostructure patterning of TiO_2 _films J Electrochem Soc 2009 156 D480 D484 10.1149/1.3216032

[B23] Lin CJ Lai YK Lin ZQ Huang JY Sun L Chen Z Controllable construction of ZnO/TiO_2 _patterning nanostructures by superhydrophilic/superhydrophobic templates New J Chem 2010 34 44 51 10.1039/b9nj00325h

[B24] Lu CH Ge WJ Hierarchical honeycomb patterns with tunable microstructures: controllable fabrication and application as replication templates Soft Matter 2011 7 2790 2796 10.1039/c0sm01159b

[B25] Sharma V Song LL Jones RL Barrow MS Williams R Srinivasarao M Effect of solvent choice on breath-figure-templated assembly of "holey" polymer films Epl-Europhys Lett 2010 91 38001 38017 10.1209/0295-5075/91/38001

[B26] Good RJ Kotsidas ED The contact angle of water on polystyrene: a study of the cause of hysteresis J Colloid Interface Sci 1978 66 360 362 10.1016/0021-9797(78)90317-X

[B27] Draper J Luzinov I Ionov L Minko S Varshney SK Stamm M Wettability and morphology of a mixed polymer brush prepared by simultaneous polymer addition Abstr Pap Am Chem S 2003 225 U623 U624

[B28] Tuteja A Choi WJ McKinley GH Cohen RE Rubner MF Design parameters for superhydrophobicity and superoleophobicity MRS Bull 2008 33 752 758 10.1557/mrs2008.161

